# Infectious factors in myocarditis: a comprehensive review of common and rare pathogens

**DOI:** 10.1186/s43044-024-00493-3

**Published:** 2024-05-24

**Authors:** Zongjie Yao, Mingjun Liang, Simin Zhu

**Affiliations:** 1 School of Health and Life Sciences, University of Health and Rehabilitation Sciences, Qindao, China; 2 Department of Intensive Care Medicine, Shanghai Six People’s Hospital Affilicated to Shanghai Jiao Tong University School of Medicine, Shanghai, China; 3 Wuhan Third Hospital-Tongren Hospital of Wuhan University, Wuhan, China

**Keywords:** Infectious myocarditis, Viruses, Prokaryotes, Parasites, Fungi

## Abstract

**Background:**

Myocarditis is a significant health threat today, with infectious agents being the most common cause. Accurate diagnosis of the etiology of infectious myocarditis is crucial for effective treatment.

**Main body:**

Infectious myocarditis can be caused by viruses, prokaryotes, parasites, and fungi. Viral infections are typically the primary cause. However, some rare opportunistic pathogens can also damage heart muscle cells in patients with immunodeficiencies, neoplasms and those who have undergone heart surgery.

**Conclusions:**

This article reviews research on common and rare pathogens of infectious myocarditis, emphasizing the complexity of its etiology, with the aim of helping clinicians make an accurate diagnosis of infectious myocarditis.

## Background

**Fig. 1:**
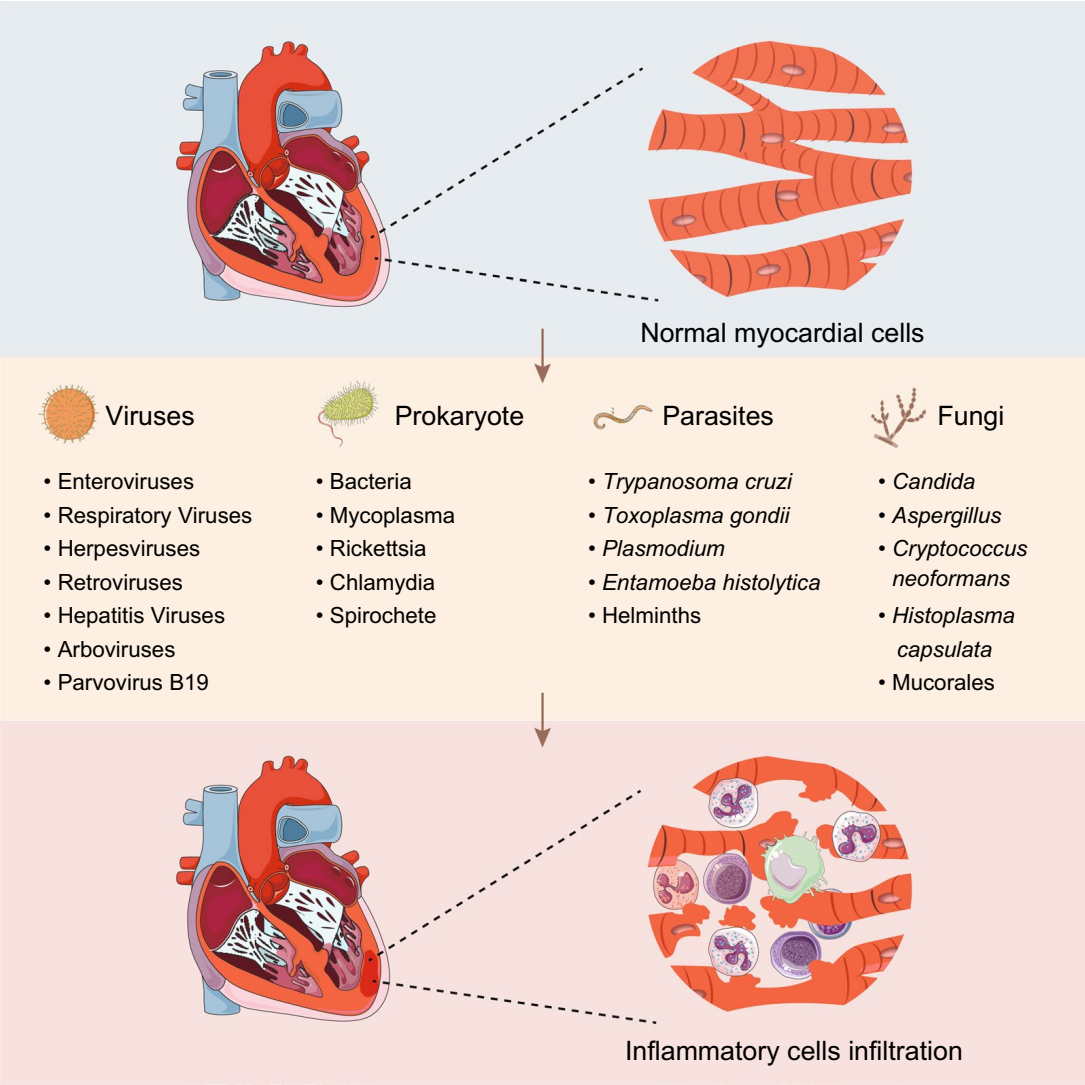
Pathogens of infectious myocarditis. Myocardial cell damage is caused by viruses, prokaryotes, parasites, and fungi, which are often accompanied by inflammatory cell infiltration

Myocarditis is an inflammatory heart disease with a diverse spectrum of clinical manifestations, from virtually asymptomatic to syncope, tachycardia, heart failure, cardiogenic shock, and even mortality [1]. Worldwide, myocarditis annually affects approximately ~ 22 in 100,000 individuals [2, 3]. Furthermore, the identification rate of it in conventional autopsies amounted to 9% [4]. Among cases of abrupt death in children, 16–21% were due to myocarditis [5]. The condition can precipitate fatal outcomes in up to 7% of young athletes [6, 7]. Myocarditis can be caused by a multitude of infectious agents (Fig. 1), such as viruses, prokaryotes, parasites, and fungi, as well as by noninfectious triggers including drugs, toxins, and hypersensitivity reactions [8]. Among these drivers, viral infection has been proved to be the most prevalent contributor to myocarditis [9, 10].

Endomyocardial biopsy (EMB) and cardiac magnetic resonance imaging (CMRI) are usually performed to diagnose myocarditis [11]. The gold standard for the diagnosis of myocarditis remains the EMB. This procedure allows the designation of “proven” myocarditis. Conversely, CMRI provides the possibility of considering “confirmed” myocarditis, offering invaluable diagnostic information in the process [12]. EMB can disclose histological and immunological signatures of myocarditis. Mononuclear cells, giant multinucleated cells, eosinophils, and other cellular infiltrates can be observable in myocarditis lesions, with myocyte loss [13]. Acute myocarditis can be lethal in short periods of time, while prolonged chronic myocarditis is always expected to result in dilated cardiomyopathy (DCM) [10, 14]. It has been estimated that between 9% and 16% of patients with myocarditis will subsequently develop DCM [8, 15, 16]. DCM is described as a severe cardiomyopathy characterized by cardiac enlargement, dilated ventricular chambers and systolic dysfunction [17]. In the absence of efficient treatment, DCM is considered an end-stage disease and sufferers often require heart transplantation.

Until the causes are identified, clinical treatments for myocarditis are usually supportive. Nevertheless, routine supportive therapy alone may not lead to favorable prognosis without effective targeting of the causes. In view of the pervasiveness of infectious myocarditis, this paper reviews the published research on commonly encountered pathogens of infectious myocarditis and also collected some evidence of atypical pathogens attacking cardiac tissue.

## Viral myocarditis

Generally, it is recognized that the pathological process of viral myocarditis can be divided into three phases: an acute phase caused by viral invasion and replication, a subacute phase characterized by inflammatory cell infiltration, and a chronic phase defined by cardiac remodeling [18]. During the acute phase, innate immune cells such as dendritic cells, natural killer cells and macrophages migrate to the heart and limit viral replication until adaptive immune response occurs. If the pathological process is not effectively addressed, it proceeds to chronic phase in which persistent low level of inflammatory response exists.

The pathogenesis of viral myocarditis includes direct injury by viral infection and consequent damage secondary to the host’s immune response [19]. Viruses commonly invade cardiomyocytes through specific receptors and co-receptors to utilize substances from the reservoir cell for their own biosynthesis and replication, which leads to metabolic disruption and even death of the host cell. Initial activation of the immune response benefits the anti-infective response by arresting viral replication. Yet, continuous and excessive immune response facilitates the development of myocarditis and promotion of DCM. Additionally, following viral infection, cardiac antigens that cross-react with viral antigens are exposed to the immune system, eliciting autoimmune responses. Autoantibody and autoreactive immune cells can result in impairment of cardiomyocytes [20].

### Enteroviruses

Nearly a quarter of viral myocarditis is attributed to enteroviruses [21]. Despite the fact that enteroviruses predominantly harbor in the gastrointestinal tract, the main diseases induced by them often exhibit symptoms outside of this area. Enterovirus genes encode multiple proteases that perform the entire viral life cycle as they process viral polyprotein into several structural and nonstructural proteins. These viral proteases also facilitate the development of disease by engaging in targeted cleavage of host cell proteins [22, 23].

#### Coxsackieviruses

Coxsackieviruses are the most common enteric virus responsible for myocarditis. The virus enters different cell types through the coxsackievirus-adenovirus receptor (CAR), a member of the family of adhesion molecules located at cell–cell junctions. Among them, Coxsackievirus B3 (CVB3) is the prevalent serotype for viral myocarditis, which binds to and aggregates decay-accelerating factor on the apical surface of cardiomyocytes, subsequently being brought to CAR at the intercalated discs and then internalized [24]. Once inside the cytoplasm, its genetic material, positive-stranded RNA, is translated into structural and nonstructural proteins. Positive-stranded RNA is transmitted through RNA-dependent RNA polymerase to produce negative-stranded RNA intermediates, which in turn generate positive-stranded RNA for packaging with structural proteins to form zygotic virus particles [25]. The zygotic virus particles are released to infect adjacent cells and thus enter the next life cycle. Acute infection of CVB3 induces intense myocarditis, while chronic infection fosters DCM. In rare instances, myocarditis caused by CVB3 can develop into DCM of an acute onset [26].

There are multiple mechanisms by which CVB3 can lead to cardiomyocyte injury or death. The nonstructural protein 2A protease of CVB3 destructs eukaryotic initiation factor 4G (eIF4G) and polyadenylate-binding proteins (PABP), preventing protein synthesis, interfering with host cell metabolism, and inducing cardiomyocyte apoptosis [27–30]. The 3C protease not only cleaves viral polyproteins but also disrupts host cell signaling proteins such as mitochondrial antiviral signaling protein (MAVS) and Toll/IL-1 receptor domain-inducing interferon-beta (TRIF), which inhibits interferon production and apoptotic signaling [31, 32].

The interaction between CVB3 and miRNAs also plays a critical role in viral pathogenesis. MiRNAs are a newly discovered class of noncoding RNAs that exert gene regulation by binding to the 3′-untranslated region (UTR) of messenger RNAs [33]. Certain miRNAs affect viral replication by directly regulating viral gene expression or host response to viral invasion [34]. During CVB3 infection, miR-141 targets the cap-binding protein eukaryotic initiation factor 4E (eIF4E) to block the translation of host proteins [35]. MiR-10a can bind to the nucleic acid sequence of CVB3 and significantly enhance viral biosynthesis [36]. Zinc finger protein-148 suppresses CVB3 replication by combining with miR-20b [37]. MiR-203 activates the protein kinase C/transcription factor AP-1 pathway to promote CVB3 survival in the early stages of infection [38]. Similarly, miR-126 facilitates viral multiplication through the extracellular regulated protein kinases 1/2 (ERK1/2) positive feedback loop and induction of glycogen synthase kinase-3β (GSK-3β) [39]. Ongoing investigations are exploring the interactions between CVB3 and several other host miRNAs [40].

A variety of other mechanisms are used by CVB3 to promote self-replication and damage cardiomyocytes. Autophagosomes fuse with lysosomes to assist Toll-like receptor 3 (TLR3) in recognizing viral material and presenting viral proteins with antigenic information via the endogenous antigen presentation pathway [41, 42]. Nevertheless, CVB3 leads to an increase in the number of autophagosomes in the host cell and prohibits their fusion with lysosomes, promoting self-survival [43, 44]. Additionally, it promotes the ubiquitin–proteasome system (UPS) of the host cell to boost replication [23, 45]. As CVB3 also activates inflammasomes, which in turn contribute to the production of inflammatory cytokines through the IL-1 signaling pathway, leading to myocardial impairment [46, 47].

#### Human parechovirus

Human parechovirus (HPEV) belongs to a subgenus of echoviruses and is originally isolated from the feces of patients with diarrhea [48]. It predominantly affects children under 1 year and is uncommon in older adolescents and adults [49]. The majority of pathogenicity studies of HPEV infections are related to HPEV-1 and HPEV-3 [50]. Besides causing slight digestive and respiratory symptoms, HPEV can also induce gross viral meningitis [51, 52]. Conventional enterovirus PCR cannot detect HPEV RNA, requiring additional HPEV testing [53]. Studies have shown that HPEV may induce myocarditis in children and adolescents [54–56]. In recent years, there have also been published papers reporting evidence of HPEV contributing to myocarditis in adults [53].

#### Reovirus

Reovirus is an envelope-less, double-stranded RNA virus that attacks central nervous tissue, liver and cardiac structures. In neonatal mice, infection through the gastrointestinal tract can result in disseminated infection and damage to the nervous, hepatic, and cardiac systems [57]. Reovirus directly inflicted significant cytopathic effects on primary cardiomyocytes of neonatal mice, ultimately leading to cell death [58, 59]. There is also evidence that reovirus is an inducer of acute myocarditis in humans [60].

### Respiratory viruses

Respiratory viruses enter the body through the respiratory tract, proliferate in the mucosal epithelium, and cause localized infection of the respiratory tract or tissue damage outside the respiratory tract. The main source of infection is patients who are infected or carrying viruses. Some respiratory viruses can be transmitted to humans from animals infected with or bearing them.

#### Novel coronavirus

The novel pathogen severe acute respiratory syndrome coronavirus 2 (SARS-CoV-2), also known as COVID-19, is similar to severe acute respiratory syndrome coronavirus (SARS-CoV) and middle east respiratory syndrome coronavirus (MERS-CoV), with the most predominant clinical manifestation being lung damage [61]. With the rapid development of relevant research, it has been found that 20–30% of patients in hospitals with symptoms of cardiovascular impairment are associated with poor clinical outcomes [62]. Clinical evidence indicates that SARS-CoV-2 can result in acute myocarditis, which can be confirmed by CMRI [63].

Angiotensin-converting enzyme 2 (ACE2) on the cell surface is the receptor for the binding of SARS-CoV-2 spike protein. ACE2 is expressed in a wide range of cells, including cardiomyocytes, fibroblasts, and endothelial cells [64]. The presence of the gene for SARS-CoV-2 was detected by PCR in cardiac tissue from autopsies of patients with COVID-19, and pathological changes of cardiomyocyte necrosis and monocyte infiltration were observed [65, 66]. Human-induced pluripotent stem cell-derived cardiomyocytes (hiPSC-CMs) and engineered myocardial tissues could be directly infected with SARS-CoV-2 [67]. In addition, the investigators reported cases of fulminant myocarditis caused by SARS-CoV-2 [68, 69]. The evidence suggests that SARS-CoV-2 may directly infect and impair myocardial tissue, which in turn induces myocarditis.

#### Adenoviruses

Adenoviruses (Adv) induce upper respiratory tract infections and pneumonia, which have also been shown to be associated with myocarditis [17, 70, 71]. In samples from patients with myocarditis, the positive rate of detection of Adv using PCR was 23% [70]. In samples obtained from DCM patients, 12% were detected [70]. Animal experiments have indicated that Adv can replicate in cardiac tissue, causing CD3^+^ T-cell-dominated inflammatory cell infiltration and excessive IFN-γ response [5]. Furthermore, persistent Adv infection is associated with progressive impairment of cardiac function and cardiac remodeling [5, 17].

#### Influenza viruses

Influenza A viruses usually result in acute respiratory infections, which can also lead to congestive heart failure and myocarditis [72, 73]. One study noted that 4.9% of patients who were hospitalized during the influenza pandemic developed cardiac complications [74]. In patients with atherosclerosis, the acute coronary syndrome could be induced by influenza A virus [75]. Mouse cardiomyocytes and hiPSC-CMs could be infected with it, resulting in damage to the cardiomyocytes. In addition, it can replicate in Purkinje cells, causing abnormal electrophysiologic activity [76].

#### Respiratory syncytial virus

Respiratory syncytial virus (RSV) is a prevalent etiology of lower respiratory tract infections in infants and young children, as well as upper respiratory tract infections in older adolescents and adults. The pathology of RSV is characterized by the induction of eosinophilic inclusion body containing multinucleated giant cells. Although extrapulmonary symptoms caused by RSV are rare, it has been demonstrated that acute myocarditis can occur [77, 78].

#### Measles virus

The measles virus causes an acute respiratory infection characterized by high fever, cough and rash. However, it can also lead to serious complications such as pneumonia and myocarditis [79–81]. The advent of the measles vaccine has been instrumental in reducing the incidence of measles epidemics. Notwithstanding, there continue to be periodic outbreaks of measles, which constitute a significant threat to child health in developing countries [82]. The study conducted in China indicates that up to 42.7% of individuals infected with measles present with symptoms of myocarditis. Furthermore, children under the age of 12 are more likely to develop this complication [83]. Given the recurrence of measles globally, particularly in regions where vaccination rates have declined, it is imperative to monitor this disease.

### Herpesviruses

#### Human herpesvirus

Human herpesvirus (HHV) is a lymphocytophilic virus that infects both endothelial and cardiomyocytes. Its nucleic acid can be incorporated into human chromosomes and transmitted vertically to offspring [84]. HHV-6 and HHV-7 have been described to initiate myocarditis [85, 86]. HHV-6 is the ubiquitous herpesvirus associated with myocarditis and predominantly infects CD4^+^ T cells [87]. Acquisition of HHV-6 infection usually occurs in childhood and can continue to be carried for life. In immunocompromised individuals, HHV-6 can be reactivated, leading to encephalitis, interstitial pneumonia and myocarditis [88]. There are two different mutant strains of HHV-6, HHV-6A and HHV-6B. HHV-6A enters cells via CD46, whereas HHV-6B via CD134 [89]. In patients with HHV-6 myocarditis, the HHV-6B genome was detected in 95% of cases, with the remainder having the HHV-6A genome [87, 90]. In spite of this, the relationship between HHV and myocarditis has not yet been entirely understood.

#### Human cytomegalovirus

Human cytomegalovirus (HCMV) is rigorously host-specific as a beta-herpesvirus which is responsible for lifelong latent infections in 50–90% of the world’s population [91]. In individuals with immunocompetent populations, it is usually asymptomatic, but reactivation can occur frequently. A study found a 3% positivity rate for HCMV detection using PCR in samples from patients with myocarditis [70]. Although HCMV has been linked to infrequent instances of acute myocarditis in adults, it can still be a life-threatening condition [92].

#### Epstein–Barr virus

Epstein–Barr virus (EBV) infection typically presents no or mild clinical symptoms in most children. In adolescents and adults, it often results in self-limiting infectious mononucleosis [93]. There is a significant association between EBV and the development of nasopharyngeal cancer and non-Hodgkin’s lymphomas in children [94]. While the primary invasion of EBV is in B lymphocytes, other systems can also be affected [95]. Myocarditis, coronary artery aneurysm, and pulmonary hypertension are disorders that can occur when the cardiovascular system is attacked by EBV [96, 97]. Several studies have shown evidence of myocarditis related to EBV [96, 98–103].

### Retrovirus

Histological evidence of co-morbid myocarditis has been observed in up to two-thirds of autopsies of untreated patients with acquired immunodeficiency syndrome (AIDS) [104]. It is known that human immunodeficiency virus (HIV) can impair cardiomyocytes, leading to localized release of cytokines and triggering infiltration of inflammatory cells [1]. AIDS patients are severely immunocompromised with significantly increased risk of comorbidities with other viral or opportunistic infections. The observation that viral particles of HIV were found in vacuoles within infected cardiomyocytes, and that nucleic acids were detected in myocardial tissue by in situ nucleic acid hybridization, suggest direct invasion of HIV into cardiomyocytes [105, 106].

### Hepatitis viruses

Hepatitis C virus (HCV) is an RNA virus that primarily affects the liver. It can also contribute to interstitial lung disease, myocarditis, DCM, and other extrahepatic manifestations [107–110]. In some patients with myocarditis and DCM, the presence of the HCV gene was detectable by EMB [107, 111]. During acute infection, HCV damages cardiomyocytes, which can lead to myocarditis. Apoptosis of cardiomyocytes is induced when they are present at persistently low levels, resulting in cardiac malfunction and even progression to DCM [107].

### Arboviruses

Zika virus (ZV) is an arbovirus which is transmitted primarily through mosquito bites, with the potential to inflict severe neurological damage, heart failure, and cardiac arrhythmias [112, 113]. Studies have demonstrated that prenatal exposure to ZV can result in congenital heart disease (CHD) in infants [114–116]. In the animal model of ZV intrauterine infection, high levels of ZV were detected in the brain and heart of mice, which presented neurological deficits [117]. Furthermore, ZV was also detected in the hearts of infected rhesus monkeys [118]. Such evidence hints that ZV can infect cardiomyocytes and induce cardiac dysfunction.

### Other viruses

Parvovirus B19 (PB19) consists of DNA viruses that spreads through the airways, gastrointestinal tract, blood, and vertical transmission, mainly affecting erythrocyte precursor cells. Infection with PB19 usually occurs in childhood and manifests itself in the form of infectious erythema. Studies have indicated that the virus can persist for life in human tissues such as the liver, skin, and tonsils [119]. Even though PB19 has a high detection rate in patients with myocarditis, it can also be found in specimens from nonmyocarditis patients [120, 121]. The source of PB19 nucleic acid is debated. Some studies have considered endothelial cells of small myocardial arteries and veins to be specific target cells for PB19 [122, 123], while one study proposed that PB19 DNA originates from mesenchymal mononuclear inflammatory cells [121]. Further investigation is required to determine the clinical relevance of PB19 to myocarditis [4].

## Prokaryotic myocarditis

Bacteria are not common causative agents in infectious myocarditis. Cases of bacterial myocarditis are usually identified at post-mortem [124]. Myocardial abscesses are typically observed in serious sepsis or bacteremia, often combined with abscess lesions in multiple organs. Bacteria may invade the myocardial tissue by directly diffusing from the endocardium or by disseminating and implanting into the myocardial tissue from purulent lesions in other tissues. Direct invasion of bacteria or secretion of toxins can cause damage to myocardial cells, which in turn can lead to inflammation of the heart, resulting in abnormal cardiac function [125]. Pathologically, bacterial myocarditis is characterized by the formation of multifocal microabscesses in the myocardial tissue, with the left ventricle most often involved [124].

### Gram-positive bacteria

#### *Staphylococcus aureus*

*Staphylococcus aureus* is generally recognized as the predominant cause of bacterial myocarditis [126]. While *S. aureus* is not a common cause of endocarditis, myocarditis with it is often the result of infection in other tissues or in immunocompromised patients [127–129]. A study reported a patient with simple *S. aureus* myocarditis presenting with symptoms of myocardial infarction who died 48 h after admission. The autopsy indicated multiple septic lesions with *S. aureus* in it [130]. However, simple *S. aureus* myocarditis is a rare occurrence in clinical practice.

#### *Streptococcus pneumoniae*

*Streptococcus pneumoniae* is the principal pathogen responsible for community-acquired pneumonia [131]. Cardiac damage can occur in invasive *S. pneumoniae* disease, whereby *S. pneumoniae* that enters the blood binds to vascular endothelial cells, which in turn attack and compromise cardiomyocytes [132]. It forms tiny lesions in the heart adjacent to blood vessels, causing abnormalities in electrophysiology and contractile function [132, 133]. *S. pneumoniae* that gains entry into cardiomyocytes develops biofilms with resistance to antibiotics [134]. Besides, biofilm-forming *S. pneumoniae* can secrete more toxin pneumolysin to kill macrophages and prevent them from recruiting neutrophils to inhibit the host immune response [134]. Still, invasive *S. pneumoniae*-induced myocarditis is not a common disease [135].

#### *Clostridium difficile*

*Clostridium difficile* is a widely distributed strictly anaerobic bacterium that can form highly resistant spores. It can cause severe clinical symptoms in patients with intestinal dysbiosis. Myocarditis caused by *C. difficile* is pathologically characterized by inflammation dominated by a neutrophilic infiltrate [136–138]. Infection with *C. difficile* in myocardial tissue is extremely infrequent, but the clinical outcome in patients is usually fatal [125].

#### *Listeria monocytogenes*

*Listeria monocytogenes* is an intracellular parasitic pathogen capable of provoking severe disease in susceptible populations [139]. In humans, *L. monocytogenes* invades the body through the gastrointestinal tract and replicates within the epithelial cells of the small intestinal mucosa. It then travels through the portal system to penetrate the liver and can also spread through the mesenteric lymph nodes and into the blood system, leading to infection of other organs [140]. Studies have identified *L. monocytogenes* that can attack cardiac tissue, leading to endocarditis, pericarditis and myocarditis [141–144].

### Gram-negative bacteria

#### *Campylobacter jejuni*

Enteritis induced by *Campylobacter jejuni* is the nearest frequent intestinal infection in developed countries with an annual incidence of 1‰ [145, 146]. In addition to intestinal symptoms, patients with *C. jejuni* infection may experience extraintestinal complications such as conjunctivitis, reactive arthritis, and Guillain-Barré syndrome [147]. Myocarditis is a rare but important consequence of *C. jejuni*, whose common symptom is chest pain secondary to diarrhea [148, 149]. Myocardial cell necrosis and inflammation with predominantly lymphocytic infiltration are the pathologic changes caused by *C. jejuni* [148]. Nonetheless, the mechanism of *C. jejuni*-induced cardiomyocyte injury requires further investigation.

#### *Legionella pneumophila*

Apart from lung involvement, *Legionella pneumophila* can be isolated and cultured in the heart, brain, and spleen in autopsy studies. Extrapulmonary airway damage from *L. pneumophila* is relatively scarce, with cardiac involvement being the most prevalent [150, 151]. Although myocarditis is an uncommon complex of *L. pneumophila* infection, the condition is usually critical [151]. Only a relatively small number of cases of myocarditis due to *L. pneumophila* infection have been reported in the literature [152, 153].

#### *Brucella*

*Brucella* can be spread to humans after direct or indirect contact with infected animals or their products, and it can affect all organs and systems [154]. The involvement of the cardiovascular system has been reported to be as high as 2% [155, 156]. Of these, endocarditis is more common, but myocarditis and pericarditis can also occur [157]. *Brucella* myocarditis is usually sensitive to antibiotics [156, 158, 159]. In *Brucella*-endemic areas, prompt consideration of diagnosing *Brucella* myocarditis and administering appropriate antibiotic therapy may prevent a poor prognosis [160].

#### *Neisseria meningitidis*

*Neisseria meningitidis* is the causative agent of meningococcal bacteremia, which can result in a mortality rate of 10–15% [161]. The most significant complications of meningococcal bacteremia are disseminated intravascular coagulation (DIC), adrenal hemorrhage, and arthritis [162]. Myocarditis is an infrequent complexity of it [163–165]. Nevertheless, in a series of autopsy studies of children who succumbed to *N. meningitidis* infection, the incidence of myocarditis ranged from 27% to 57% [166, 167].

#### Gonococcus

Disseminated gonococcal infections are relatively rare occurrences in *Neisseria gonorrhoeae*. Cardiac involvement by gonococci often carries poor prognostic implications. Prior to 1940s, gonococcal endocarditis accounted for 11–26% of all cases of bacterial endocarditis, with myocarditis being exceedingly infrequent [168, 169]. With the advent of antibiotic therapy, the incidence of cardiac complications due to gonococci declined significantly. While, as HIV infection aggravates, gonococcal-induced myocarditis may reappear [170].

#### *Salmonella*

*Salmonella* can cause a variety of diseases, including enteric fever, typhoid, and diarrhea [171]. It is an unusual agent in myocarditis [172]. *Salmonella typhi* and *Salmonella paratyphi* are the main pathogens in the reported cases of *Salmonella* myocarditis. In contrast, nontyphoidal *Salmonella* myocarditis occurs frequently in young Western men and has a dismal outcome [173–175].

### *Mycoplasma pneumoniae*

*Mycoplasma pneumoniae* gives rise to primary atypical pneumonia, tracheobronchitis, pharyngitis, and asthma in humans. Extrapulmonary complexes arise at various times after the onset of infection, even in asymptomatic infections. Up to a quarter of *M. pneumoniae* infections can develop extrapulmonary symptoms [176]. Cardiac complications are believed to occur slightly more frequently in adults than in children [176, 177]. Cardiac involvement by *M. pneumoniae* can present with a spectrum of symptoms varying from asymptomatic to severe chest pain [178, 179]. There is, however, a good response of mycoplasma to macrolide antibiotics, and timely diagnosis and treatment have a good outcome.

### Rickettsia

Transmission of rickettsia to humans via arthropod bites elicits the typical triad of fever, headache, and rash [180]. Microangiitis is the hallmark pathological change associated with rickettsia. Myocarditis is a unusual complication of rickettsia and often appears in acute infections with rickettsia [181, 182].

### *Chlamydia pneumoniae*

*Chlamydia pneumoniae* is a widespread pathogen in humans and frequently responsible for a large range of infectious diseases. Previous reports have described the involvement of *C. pneumoniae* in heart-related conditions [183]. *C. pneumoniae* particles are commonly seen in atherosclerotic plaques and are considered to be associated with atherosclerosis [184]. Additionally, studies have indicated a correlation between *C. pneumoniae* and abdominal aortic aneurysms as well as valvular disease [184]. Myocarditis, endocarditis, and pericarditis can be observed in patients with *C. pneumoniae* infection in clinical settings [185].

### Spirochete

Lyme disease is caused by *Borrelia burgdorferi* infection. Impaired heart function often presents as atrioventricular block in Lyme disease [186]. A history of travel to Lyme disease-endemic areas or tick bites should raise suspicion of Lyme disease myocarditis when patients present with wandering erythema and abnormal cardiac function [187]. Symptoms of myocarditis due to Lyme disease are generally mild, transient, and somewhat self-limiting [188]. If recognized and treated early, the prognosis is often favorable [189, 190].

## Parasitic myocarditis

### Protozoa

#### *Trypanosoma cruzi*

In Central and South America, *Trypanosoma cruzi* is the main causative agent of myocarditis [191]. In humans, infection with the intracellular protozoan *T. cruzi* often leads to Chagas disease. Although acute infections with *T. cruzi* presenting with overt signs of myocarditis are uncommon in the clinic, chronic infections often give rise to cardiomyopathy, gastrointestinal disorders, and other organ injuries [192, 193]. Approximately 20–30% of chronic infections may develop cardiomyopathy with symptoms of arrhythmias, systolic dysfunction, and heart failure [193–195]. *T. cruzi* myocarditis is marked by focal inflammation, cardiomyocyte lysis, necrosis, and progressive fibrosis of the heart, with lesions located primarily in the apical and basal portions of the posterior and inferior walls of the heart [193, 196]. The inflammatory cells infiltrated as a result of *T. cruzi* are initially dominated by neutrophils and macrophages, which are later accompanied by the addition of lymphocytes and eosinophils [197]. The clinical outcome of the disease depends on the virulence of the *T. cruzi* strain and the genetic predisposition of the infected individual. However, the mechanisms by which *T. cruzi* induce cardiomyopathy are still being explored.

#### *Toxoplasma gondii*

*Toxoplasma gondii* infection is prevalent in tropical and subtropical areas with poor sanitary conditions. Patients are often infected through contact with contaminated meat, water, or infected felines [198]. In immunocompetent infected individuals, there are often no obvious symptoms. But *T. gondii* can attack organs such as the brain, heart, and lungs in immunocompromised persons. Of these, the central nervous system is engaged for the most part [199]. Myocarditis caused by *T. gondii* is more usual in patients with AIDS [200, 201].

#### *Plasmodium*

Malaria is among the most prevalent parasitic diseases in the tropics. It presents with vague symptoms such as fever, chills, and headache [202]. *Plasmodium falciparum* and *Plasmodium intertrigo* are responsible for the majority of cases of cardiac complications. A meta-study revealed a 7% prevalence of cardiovascular complications among adult patients with symptomatic malaria [203]. Myocarditis and acute coronary syndromes had the highest prevalence of cardiovascular complications [203].

#### *Entamoeba histolytica*

Amoebiasis is triggered by *Entamoeba histolytica* and endemic mainly in tropical developing countries [204, 205]. Following malaria and schistosomiasis, amoebiasis is the third most frequent cause of parasite-induced mortality [205]. Parenteral amebiasis primarily affects the liver [206]. Amebic pericarditis is a relatively uncommon but potentially serious complication of abscesses in the liver, which is observed in the pediatric population [205, 207]. And myocarditis caused by *E. histolytica* is considerably rarer [208].

### Helminths

As public health improves, helminth infections are becoming less widespread. Helminths can damage the heart in the form of eggs, larvae or adults, leading to various cardiac pathologies. For instance, larvae of worms such as the *Taenia solium*, *Trichinella spiralis*, and *Echinococcus granulosus* can invade the heart via the blood circulation or the lymphatic system [209]. Myocarditis induced by helminths is typically characterized by eosinophilic infiltration, although clinical manifestations can vary significantly [209].

## Fungal myocarditis

Fungal infections usually occur in individuals with severe immune compromise, such as those undergoing intensive corticosteroid treatment, those with neoplasia, or those with AIDS. Fungal myocarditis arises in disseminated fungal infections, which are often only detected post-mortem [210].

### *Candida*

*Candida* is a fellow of resident oral and cutaneous flora that may invade several organs, including the heart, under certain conditions [211]. The closest pathogen to fungal myocarditis is *Candida*. The incidence of myocarditis ranges from 10% to 60% in patients with the presence of disseminated *Candida* [210]. *Candida* myocarditis can be observed as white nodular foci in myocardial tissue in which light microscopy reveals necrotic cardiomyocytes and infiltrating inflammatory cells with pseudohyphae and oval bodies [212, 213].

### *Aspergillus*

Invasive aspergillosis takes place in immunocompromised or postoperative cardiac patients [214, 215]. *Aspergillus* that penetrates the pulmonary vasculature can infect the endocardium upstream, which in turn invades and harms the myocardium [210]. Light microscopy of *Aspergillus* lesion tissue discloses bouquets or branched mycelium [216]. Blood cultures are generally negative in patients with *Aspergillus* myocarditis, oftentimes along with a high mortality rate [217].

### *Cryptococcus neoformans*

*Cryptococcus neoformans* is an opportunistically pathogen, but its culture positivity rate increases with the growing number of AIDS patients. About 5–10% of patients with AIDS may have *C. neoformans* isolated [218, 219]. The combination of *C. neoformans* myocarditis in patients with AIDS is often accompanied by poor clinical outcomes [220]. It has been demonstrated in animal studies that *C. neoformans* may produce focal necrosis of the myocardium with inflammatory process accompanied by lymphocyte and macrophage infiltration [221].

### *Histoplasma capsulata*

The inhalation of *Histoplasma capsulatum* spores present in soil may result in a mild pulmonary burden. This is particularly prevalent in the central and eastern United States [222]. Among patients suffering from immunocompromised conditions, *H. capsulatum* has the potential to invade multiple organs, resulting in cases of pericarditis and endocarditis [223]. Myocarditis due to *H. capsulatum* remains extremely scarce, with only a few cases having been described [224].

### Mucorales

Mucormycosis tends to be more epidemic in developing countries, mainly in impaired immunocompetent individuals [225]. Mucorales constitutes 3–13% of fungal infections in autopsy studies [225, 226]. Once the fungus enters the bloodstream, it forms thrombi, which can cause tissue necrosis. The sinuses, lungs, and skin are the most commonly affected sites in mucormycosis [227]. Disseminated mucormycosis has been demonstrated to affect the endocardium and myocardium, resulting in damage [228, 229]. The diagnosis of mucormycosis remains challenging due to the lack of specific serum biomarkers and low culture positivity, with extremely negative consequences [229].

## Conclusions

Infectious myocarditis is motivated by what appears to be the combination of pathogen-induced myocardial cell injury and host immune responses. Despite being a significant public health risk, there are still many mysteries surrounding the causative factors of pathogens and host mechanisms. Early recognition and determination of the etiology are crucial for patient prognosis. Timely and effective interventions are necessary to prevent poor clinical outcomes. This review discusses the complex etiology of pathogen-induced myocarditis. Clinicians should be aware of the rare pathogens associated with myocarditis to make an accurate diagnosis.

## Data Availability

Not applicable.

## Abbreviations

EMB: Endomyocardial biopsy
DCM: Dilated cardiomyopathy
CVB3: Coxsackievirus B3
CAR: Coxsackievirus-adenovirus receptor
HPEV: Human parechovirus
ACE2: Angiotensin-converting enzyme 2
hiPSC-CMs: Human-induced pluripotent stem cell-derived cardiomyocytes
Adv: Adenoviruses
RSV: Respiratory syncytial virus
HHV: Human herpesvirus
HCMV: Human cytomegalovirus
EBV: Epstein–Barr virus
AIDS: Acquired immunodeficiency syndrome
HIV: Human immunodeficiency virus
HCV: Hepatitis C virus
ZV: Zika virus
PB19: Parvovirus B19
DIC: Disseminated intravascular coagulation
